# Lifestyle behaviours of Lebanese-Australians: Cross-sectional findings from The 45 and Up Study

**DOI:** 10.1371/journal.pone.0181217

**Published:** 2017-07-13

**Authors:** Aymen El Masri, Gregory S. Kolt, Thomas Astell-Burt, Emma S. George

**Affiliations:** 1 School of Science and Health, Western Sydney University, Sydney, New South Wales, Australia; 2 Population Wellbeing and Environment Research Lab (PowerLab), School of Health and Society, Faculty of Social Sciences, University of Wollongong, Wollongong, New South Wales, Australia; 3 Early Start Research Institute, Faculty of Social Sciences, University of Wollongong, Wollongong, New South Wales, Australia; Macquarie University Faculty of Human Sciences, AUSTRALIA

## Abstract

Little is known regarding the health and lifestyle behaviours of Australians of Lebanese ethnicity. The available evidence suggests that Australians of Lebanese ethnicity who were born in Lebanon reportedly have higher rates of cardiovascular disease-related and type 2 diabetes-related complications when compared with the wider Australian population. The aim of this study is to compare lifestyle behaviours of middle-aged to older adults of Lebanese ethnicity born in Lebanon, Australia, and elsewhere to those of Australian ethnicity. Participants were 37,419 Australians aged ≥45 years, from the baseline dataset of The 45 and Up Study which included 4 groups of interest: those of Australian ethnicity (n = 36,707) [Reference]; those of Lebanese ethnicity born in Lebanon (n = 346); 302 those of Lebanese ethnicity born in Australia (n = 302); and those of Lebanese ethnicity born elsewhere (n = 64). Multilevel logistic regression was used to examine the odds of those of Lebanese ethnicity reporting suboptimal lifestyle behaviours (insufficient physical activity, prolonged sitting, smoking, sleep duration, and various diet-related behaviours) relative to those of Australian ethnicity. Multilevel linear regression was used to examine the clustering of suboptimal lifestyle behaviours through a ‘lifestyle index’ score ranging from 0–9 (sum of all lifestyle behaviours for each subject). The lifestyle index score was lower among Lebanese-born (-0.36, 95% CI -0.51, -0.22, *p*<0.001) and Australian-born (-0.17, 95% CI -0.32, -0.02, *p* = 0.031) people of Lebanese ethnicity in comparison to those of Australian ethnicity. Those of Lebanese ethnicity born in Lebanon had higher odds of reporting suboptimal lifestyle behaviours for physical activity, smoking, and sleep duration, and lower odds of reporting optimal lifestyle behaviours for sitting time, fruit, processed meat, and alcohol consumption, when compared with those of Australian ethnicity. Differences in the individual lifestyle behaviours for those of Lebanese ethnicity born in Australia and elsewhere compared with those of Australian ethnicity were fewer. Lifestyle behaviours of those of Lebanese ethnicity vary by country of birth and a lower level of suboptimal lifestyle behaviour clustering was apparent among Lebanese-born and Australian-born middle-aged to older adults of Lebanese ethnicity.

## Introduction

Lower levels of physical activity (PA) [1, 2], sedentary behaviour [3], poor diet [4], smoking [2, 5], and suboptimal sleep durations [6] are all associated with poor health. When suboptimal lifestyle behaviours cluster, however, there is an increased risk of poorer health [7] and all-cause mortality [8]. Recommended guidelines for many of these lifestyle behaviours were developed based upon empirical evidence [9–12]. Addressing these lifestyle factors has been the focus of many health promotion initiatives and campaigns in Australia, such as the Healthy Communities Initiative [13] and the National Tobacco Campaign [14].

Australia is renowned as a culturally and linguistically diverse (CALD) country and this is demonstrated by the fact that 26% of the population reported being born overseas in the 2011 Census [15]. The ‘healthy migrant effect’ suggests that migrants have good and oftentimes better health than the host population, as immigration is usually only carried out by those with the health capacity to endure the associated stressors of such a move [16]. This hypothesis, however, does not apply to all migrants (e.g., refugees) [17, 18]. With increased duration of residence in the host country it is suggested that this initial health advantage diminishes as certain health behaviours of the host population are adopted by migrants, a process known as acculturation [16]. Previous research, however, has shown that the ‘healthy migrant effect’ [18] and the convergence of health towards that of the host population [19] does not occur for all CALD populations. Furthermore, the lifestyle behaviours of later generation CALD populations do not always necessarily converge with those of the host population [20, 21].

One of the largest CALD groups within the Australian context are people of Lebanese ethnicity. Lebanese migration to Australia occurred over three main waves in 1880–1947, 1947–1975, and following the Lebanese civil war in 1975 [22, 23]. Immigration levels of Lebanese people to Australia peaked following the latter period [24], which was mainly due to the civil war [23]. In 2011, the Lebanese population in Australia was approximately 203,139 [22], and the vast majority (approximately 72%) resided in Australia’s most populous state, New South Wales (NSW) [25]. The evidence base on the health of Australians of Lebanese ethnicity is limited, and the available evidence focuses only on those born in Lebanon. It is reported that Lebanese-born adults residing in Australia experience higher rates of cardiovascular disease-related complications and type 2 diabetes mellitus (T2DM)-related health complications when compared with Australian-born adults [26]. Further, it has been reported that Lebanese-born adults residing in Australia experienced higher incidence rates of various types of cancers, such as colon and breast cancer, in comparison to their counterparts in Lebanon, yet mostly lower rates in comparison to Australian-born adults [27]. In different migratory contexts, the prevalence of T2DM among Lebanese immigrants to Denmark was higher when compared with the native Danish population [28]. These chronic diseases are all lifestyle-related and are largely preventable with healthier lifestyle choices.

In the Lebanese context, dietary factors, tobacco smoking, and low levels of PA were in the top 10 risk factors attributable to mortality and disability in 2015 (ranked 1^st^, 5^th^, and 7^th^, respectively) [29]. The prevalence of current tobacco smokers among Lebanese adults in Lebanon aged 25–64 was 38.5% in 2009, 59% reportedly did not consume alcohol within the previous 12 months, and 45.8% were not engaging in sufficient levels of PA [30]. Moreover, 34.1% had between 3–4 chronic disease risk factors (daily smoking, low level of PA, overweight or obese, high blood pressure) and 55.6% had 1–2 of these chronic disease risk factors [30].

The evidence to date on the lifestyle behaviours of Australians of Lebanese ethnicity focuses on those born in Lebanon and indicates that they have a higher prevalence of suboptimal lifestyle behaviours such as insufficient PA, smoking, and insufficient vegetable consumption, relative to the Australian-born population [31, 32]. Furthermore, it is reported that the prevalence of CVD risk factors, such as insufficient PA, are reportedly high among those of Lebanese ethnicity [33]. Little is known, however, about the potential differences or similarities in lifestyle behaviours of those of Lebanese ethnicity by country of birth (i.e., Lebanese-born, Australian-born, or born in other countries) relative to those of Australian ethnicity. Such comparisons will provide a unique insight, as it has been reported that differences in health among ethnic groups from the same origin differ by country of birth [34, 35]. This information can be used to guide the development of health promotion initiatives and interventions targeting this population. Moreover, this could also incite further research examining the lifestyle behaviours of Australians of Lebanese ethnicity and their contribution towards lifestyle-related chronic disease risk. The aim of this study is to examine the odds and clustering of suboptimal lifestyle behaviours among Australians of Lebanese ethnicity born in Lebanon, Australia, or elsewhere in comparison to those of Australian ethnicity.

## Methods

### The 45 and Up Study

Baseline data were drawn from The Sax Institute’s 45 and Up Study, a large cohort study of approximately 267,000 adults aged 45 years and older resident in NSW, Australia. The 45 and Up Study has been described in detail elsewhere [36]. Briefly, participants were randomly sampled from the Department of Human Services (formerly Medicare Australia) enrolment database and were recruited between February 2006 and December 2009. Participants were mailed a gender-specific self-report questionnaire, information leaflet, consent form, and a reply paid envelope and were required to give their written informed consent for participation (The questionnaire can be found using the following link: https://www.saxinstitute.org.au/our-work/45-up-study/questionnaires/). The 45 and Up Study has an estimated response rate of 18%, however, estimates from the 45 and Up Study are consistent with population-based studies with more representative samples and higher response rates [37]. The 45 and Up Study was approved by the University of NSW Human Ethics Committee. Reciprocal ethics approval was obtained from the Western Sydney University Human Research Ethics Committee (H10930).

### Participants

Participants for this study were a subset from baseline data of The 45 and Up Study and were identified based on their responses to the questions “In which country were you born in?” and “What is your ancestry?”. The question “What is your ancestry?” is an indicator of ethnicity and is used in the Australian Census (Participants had the option to select up to 2 ancestries). These questions were used to identify four groups of interest: (1) people of Australian ethnicity (i.e., those who reported Australian ancestry only and were born in Australia); (2) people of Lebanese ethnicity (i.e., those who reported Lebanese ancestry and currently reside in Australia) and were born in Lebanon; (3) people of Lebanese ethnicity and were born in Australia; and (4) people of Lebanese ethnicity and were born elsewhere. To retain the greatest number of participants of Lebanese ethnicity in the current sample, all participants who reported Lebanese ancestry, either alone, or in addition to another ancestry, were included in the sample and stratified by country of birth. Exclusions were made for those with an invalid lifestyle index (i.e., scores with missing data on at least one lifestyle behaviour), missing a Statistical Area Level 2, and other socio-demographic variables (Refer to Figure in S1 Fig for participant flow). The final sample used in the analyses of this study were 37,419 men and women aged 45 and older, which comprised of those who were of Australian ethnicity (n = 36,707), Lebanese ethnicity born in Lebanon (n = 346), Lebanese ethnicity born in Australia (n = 302), and Lebanese ethnicity born elsewhere (n = 64). As the survey was only available in English [36], Australians of Lebanese ethnicity with limited English proficiency may not have been able to complete the survey. Although those of Lebanese ethnicity only comprised 1.9% of this study sample, this sample size is comparable to the Lebanese population as a whole in NSW as of 2011 (146, 872, approximately 2.1% of the NSW population) [25].

### Lifestyle behaviours

A range of lifestyle behaviours were examined individually and included in the calculation of the ‘lifestyle index’. The ‘lifestyle index’ score ranged from 0–9 and is the summed total of suboptimal lifestyle behaviours that an individual reported. A lifestyle index has been similarly used in previous epidemiological studies [7, 8, 32, 38–40], however, making conclusions on the health of a population based on this approach alone masks important information [32]. Consequently, concurrent examination of individual lifestyle behaviours is needed to provide further information [32, 41]. Lifestyle behaviours were dichotomised in accordance with published health guidelines (where possible), and suboptimal lifestyle behaviours coded ‘1’ and the guideline or recommended lifestyle behaviours coded ‘0’.

#### Physical activity

PA was assessed in The 45 and Up Study using items from the Active Australia Survey [42], which has been reported to be valid [43] and reliable [44]. In accordance with Active Australia Survey guidelines, sufficient PA was defined as accumulating at least 150 minutes of PA/week (coded ‘0’) [42] whereas insufficient PA was defined as accumulating less than 150 minutes of PA/week (coded ‘1’).

#### Sitting time

Information on the number of hours spent sitting each 24-hour day was requested, which is similar to the assessment utilised in the validated and reliable International Physical Activity Questionnaire [45]. Prolonged sitting was dichotomised as sitting for >7 hours/day (coded ‘1’) and ≤7 hours/day (coded ‘0’), which is in accordance with a recent meta-analysis linking sitting for >7 hours/day with an increased risk of all-cause mortality [46].

#### Tobacco smoking

Smoking was based on current smoking status, with current smokers coded ‘1’ and non-smokers (including ex-smokers) coded ‘0’.

#### Alcohol consumption

Information on the number of standard drinks consumed each week was requested. Alcohol consumption was dichotomised in accordance with recommended guidelines, which were >14 drinks/week (coded ‘1’) and ≤14 drinks/week (coded ‘0’) [11]. This has been used in a previous study [39].

#### Vegetable consumption

Total vegetable consumption was based on the sum of raw and cooked vegetables consumed each day and was categorised as ≥5 servings/day (coded ‘0’) and <5 servings/day (coded ‘1’) in accordance with Australian national guidelines [12].

#### Fruit consumption

Similarly, information on the total number of servings of fruit consumed each day was requested. In accordance with Australian national guidelines [12], fruit consumption was categorised as ≥2 servings/day (coded ‘0’) and <2 servings/day (coded ‘1’).

#### Processed meat consumption

Information on the number of times processed meat was consumed each week was requested. In accordance with recent evidence [47], those who reported any processed meat consumption (coded ‘1’) were distinguished from those who reported no processed meat consumption (coded ‘0’).

#### Red meat consumption

Participants were also asked to report the number of times they consumed red meat each week. As an approximation of the Cancer Council Australia’s [9] recommendation of consuming approximately 2 serves of red meat 3–4 times/week, those consuming red meat 3–4 times/week were within recommended guidelines and coded ‘0’, whereas those consuming <3 or >4 times/week were not within recommended guidelines and coded ‘1’. A limitation of using this categorisation is that those who follow vegan or vegetarian diets would be placed in the ‘suboptimal’ category.

#### Sleep duration

Participants reported the number of hours slept during each 24-hour day. Time spent sleeping was categorised as 7–9 hours/day (coded ‘0’) and <7 or >9 hours/day (coded ‘1’), which is based on the findings of a meta-analysis linking shorter and longer sleep durations with deleterious outcomes [6].

#### Lifestyle index

The lifestyle index was calculated by summing the total of the 9 lifestyle behaviours with a score ranging from 0–9, with the higher scores indicating a greater number of suboptimal lifestyle behaviours.

### Other variables

Age was categorised as 45–54, 55–64, 65–74, and ≥75 years. Participants indicated their level of education which included no qualifications, school or intermediate certificate, higher school certificate, trade/apprenticeship, certificate/diploma, and university degree or higher (responses to trade/apprenticeship and certificate/diploma were aggregated due to observations of <5 in these categories). Marital status was dichotomised as couple (married, de facto/living with a partner) or single (single, widowed, divorced, or separated). Employment status was categorised as employed (fulltime, part-time, self-employed) and unemployed/unpaid (retired/pensioner, partially retired, disabled/sick, unpaid work, studying, look after home/family, unemployed).

Neighbourhood affluence was accounted for using the Socio-Economic Index for Areas (SEIFA), ‘Index of Relative Socio-Economic Advantage/Disadvantage’ [48]. This index was categorised into quartiles, with the lowest quartile representing the most disadvantaged, and the higher scores representing the most advantaged. Geographical remoteness of residence was accounted for using the ‘Accessibility/Remoteness Index of Australia’ [49]. The Accessibility/Remoteness Index (ARIA) was dichotomised to differentiate between those living in major cities (Index = ≤0.2) from regional and remote residents (Index = >0.2).

### Statistical analyses

Descriptive statistics were used to examine the distribution of socio-demographic, lifestyle behaviours, and the lifestyle index across the sample. Chi-squared statistics were conducted to examine differences in the socio-demographic and lifestyle behaviours across the four respective groups. To account for the potential clustering of participants in ethnic enclaves, multilevel models were utilised. Participants were nested within Statistical Area Level 2 derived from postcode, which are used to represent communities, ranging from 3,000 to 25,000 persons, and comprise of approximately 10,000 persons on average [50]. Multilevel logistic regression models were conducted for each suboptimal lifestyle behaviour (i.e., insufficient PA, prolonged sitting, smoking, excessive alcohol consumption, insufficient vegetable and fruit consumption, processed and red meat consumption, and sleep duration) to examine if there were any key differences among those of Lebanese ethnicity born in Lebanon, Australia, and elsewhere in comparison to those of Australian ethnicity. Multilevel linear regression was conducted in order to examine the difference in the lifestyle index score among those of Lebanese ethnicity in comparison to those of Australian ethnicity.

Results included an unadjusted model (Model 1) and sequential adjustment was made for a range of socio-demographic variables: age and gender (Model 2), followed by education, employment status, marital status (Model 3), and final adjustments were made for the Socio-economic Index for Areas and Accessibility/Remoteness Index of Australia (Model 4). Exclusions were made for those with an invalid lifestyle index (i.e., scores with missing data on at least one lifestyle behaviour) (n = 18,032). Further exclusions were made for those missing a Statistical Area Level 2 (n = 7,048) and then on other independent variables, education, marital status, employment, Socio-Economic Index for Areas, and the Accessibility/Remoteness Index of Australia (n = 901). Data-analysis was performed in STATA 12.0 (StataCorp, College Station, TX USA) and all analyses are presented with 95% Confidence Intervals (CI).

## Results

Table 1 presents the demographic characteristics and lifestyle behaviour distribution among the sample. The mean (±SD) lifestyle index score was 3.49/9 (±1.42) for those of Australian ethnicity, 3.34/9 (±1.37) for those of Lebanese ethnicity born in Lebanon, 3.30/9 (±1.44) for those of Lebanese ethnicity born in Australia, and 3.48/9 (±1.37) for those of Lebanese ethnicity born elsewhere. There were statistically significant differences for age (χ^2^ = 33.75, df = 9, *p*<0.001), gender (χ^2^ = 17.97, df = 3, *p*<0.001), education (χ^2^ = 226.22, df = 12, *p*<0.001), employment status (χ^2^ = 32.97, df = 3, *p*<0.001), SEIFA (χ^2^ = 51.46, df = 9, *p*<0.001), ARIA (χ^2^ = 303.42, df = 3, *p*<0.001), PA (χ^2^ = 81.29, df = 3, *p*<0.001), smoking status (χ^2^ = 84.43, df = 3, *p*<0.001), alcohol consumption (χ^2^ = 72.49, df = 3, *p*<0.001), fruit consumption (χ^2^ = 34.60, df = 3, *p*<0.001), processed meat consumption (χ^2^ = 142.36, df = 3, *p*<0.001), and sleep duration (χ^2^ = 43.86, df = 3, *p*<0.001) between the four respective groups.

**Table 1:** Sample characteristics.

	Australian ethnicity (n = 36,707)		Lebanese ethnicity						Chi-squared
			Lebanese-born (n = 346)		Australian-born (n = 302)		Born in other countries (n = 64)		
**Mean lifestyle index score**	3.49 (±1.42)		3.34 (±1.37)		3.30 (±1.44)		3.48 (±1.37)		
**Age**									
45–54	12,733	34.7%	142	41.0%	143	47.4%	20	31.3%	χ^2^ = 33.75, df = 9, *p*<0.001
55–64	12,427	33.9%	114	32.9%	93	30.8%	28	43.8%	
65–74	7,170	19.5%	54	15.6%	41	13.6%	9	14.1%	
≥75	4,377	11.9%	36	10.4%	25	8.3%	7	10.9%	
**Gender**									
Male	18,881	51.4%	203	58.7%	127	42.1%	34	53.1%	χ^2^ = 17.97, df = 3, *p*<0.001
Female	17,826	48.6%	143	41.3%	175	57.9%	30	46.9%	
**Education**									
No education	3,882	10.6%	96	27.7%	16	5.3%	5	7.8%	χ^2^ = 226.22, df = 12, *p*<0.001
School certificate	8,820	24.0%	76	22.0%	69	22.8%	10	15.6%	
Higher school certificate	3,020	8.2%	68	19.7%	33	10.9%	13	20.3%	
Certificate/Diploma/Apprenticeship	11,772	32.1%	65	18.8%	100	33.1%	11	17.2%	
University	9,213	25.1%	41	11.8%	84	27.8%	25	39.1%	
**Marital status**									
Single	7,508	20.5%	69	19.9%	81	26.8%	14	21.9%	χ^2^ = 7.59, df = 3, *p =* 0.055
In a relationship	29,199	79.5%	277	80.1%	221	73.2%	50	78.1%	
**Employment status**									
unemployed	17,407	47.4%	201	58.1%	108	35.8%	27	42.2%	χ^2^ = 32.97, df = 3, *p*<0.001
employed	19,300	52.6%	145	41.9%	194	64.2%	37	57.8%	
**SEIFA**									
Quartile 1	9,014	24.6%	123	35.5%	65	21.5%	9	14.1%	χ^2^ = 51.46, df = 9, *p*<0.001
Quartile 2	9,205	25.1%	102	29.5%	72	23.8%	13	20.3%	
Quartile 3	9,098	24.8%	74	21.4%	73	24.2%	22	34.4%	
Quartile 4	9,390	25.6%	47	13.6%	92	30.5%	20	31.3%	
**ARIA**									
Major cities	18,827	51.3%	326	94.2%	186	61.6%	58	90.6%	χ^2^ = 303.42, df = 3, *p*<0.001
Rural/remote	17,880	48.7%	20	5.8%	116	38.4%	6	9.4%	
**Year of arrival**									
pre 1975	n/a	n/a	181	56.0%	n/a	n/a	35	57.0%	
1975–1990	n/a	n/a	118	36.0%	n/a	n/a	19	3.0%	
1991–2008	n/a	n/a	25	8.0%	n/a	n/a	7	11.0%	
**Physical activity**									
≥150 minutes/week	29,235	79.6%	214	61.8%	253	83.8%	40	62.5%	χ^2^ = 81.29, df = 3, *p*<0.001
<150 minutes/week	7,472	20.4%	132	38.2%	49	16.2%	24	37.5%	
**Sitting time**									
≤7 hours/d	27,061	73.7%	275	79.5%	229	75.8%	48	75.0%	χ^2^ = 6.58, df = 3, *p =* 0.870
>7 hours/d	9,646	26.3%	71	20.5%	73	24.2%	16	25.0%	
**Smoking status**									
non-smoker	34,095	92.9%	277	80.1%	277	91.7%	59	92.2%	χ^2^ = 84.43, df = 3, *p*<0.001
smoker	2,612	7.1%	69	19.9%	25	8.3%	5	7.8%	
**Alcohol consumption**									
≤14 drinks/week	30,369	82.7%	338	97.7%	277	91.7%	57	89.1%	χ^2^ = 72.49, df = 3, *p*<0.001
>14 drinks/week	6,338	17.3%	8	2.3%	25	8.3%	7	10.9%	
**Vegetable consumption**									
≥5 serves/day	7,932	21.6%	63	18.2%	69	22.8%	9	14.1%	χ^2^ = 4.77, df = 3, *p =* 0.190
<5 serves/day	28,775	78.4%	283	81.8%	233	77.2%	55	85.9%	
**Fruit consumption**									
≥2 serves/day	20,675	56.3%	238	68.8%	193	63.9%	46	71.9%	χ^2^ = 34.60, df = 3, *p*<0.001
<2 serves/day	16,032	43.7%	108	31.2%	109	36.1%	18	28.1%	
**Processed meat consumption**									
No processed meat	7,860	21.4%	165	47.7%	76	25.2%	17	26.6%	χ^2^ = 142.36, df = 3, *p*<0.001
At least once/week	28,847	78.6%	181	52.3%	226	74.8%	47	73.4%	
**Red meat consumption**									
3–4 times/week	16,182	44.1%	162	46.8%	130	43.0%	28	43.8%	χ^2^ = 1.18, df = 3, *p =* 0.757
<3 or >4 times/week	20,525	55.9%	184	53.2%	172	57.0%	36	56.3%	
**Sleep duration**									
7–9 hours/day	28,829	78.5%	225	65.0%	218	72.2%	49	76.6%	χ^2^ = 43.86, df = 3, *p*<0.001
<7 or >9 hours/day	7,878	21.5%	121	35.0%	84	27.8%	15	23.4%	

***Note***. SEIFA—Socio-Economic Index for Areas; ARIA—Accessibility/Remoteness Index of Australia

Table 2 presents the results for the individual lifestyle behaviour models. In comparison to those of Australian ethnicity, the Lebanese-born Lebanese ethnic group had higher odds of reporting not meeting PA guidelines, smoking, and suboptimal sleep durations, and had lower odds of reporting sitting for >7 hours per day, insufficient fruit consumption, processed meat consumption, and excessive alcohol consumption. The Australian-born Lebanese ethnic group had higher odds of reporting suboptimal sleep durations, and had lower odds of reporting insufficient fruit consumption and excessive alcohol consumption when compared with those of Australian ethnicity. The Lebanese ethnic group born elsewhere had higher odds of reporting not meeting PA guidelines and had lower odds of reporting insufficient fruit consumption when compared with those of Australian ethnicity.

**Table 2:** Odds ratios (95% CI) for reporting suboptimal lifestyle behaviours among those of Lebanese ethnicity relative to those of Australian ethnicity.

	Australian ethnicity (ref.)	Lebanese ethnicity		
		Lebanese-born	Australian-born	Born in other countries
Physical activity				
Model 1^a^	1.00	2.34 (1.87, 2.93) *p*<0.001	0.75 (0.55, 1.02) *p* = 0.063	2.30 (1.38, 3.83) *p* = 0.001
Model 2^b^	1.00	2.42 (1.93, 3.04) *p*<0.001	0.77 (0.56, 1.05) *p* = 0.096	2.38 (1.42, 3.98) *p =* 0.001
Model 3^c^	1.00	2.20 (1.75, 2.76) *p*<0.001	0.78 (0.57, 1.06) *p =* 0.115	2.52 (1.50, 4.22) *p*<0.001
Model 4^d^	1.00	2.03 (1.62, 2.55) *p*<0.001	0.78 (0.57, 1.06) *p =* 0.115	2.44 (1.45, 4.09) *p =* 0.001
ICC for Model 1: 0.014 (0.010, 0.021)				
Sitting time				
Model 1^a^	1.00	0.65 (0.49, 0.85) *p =* 0.002	0.85 (0.65, 1.11) *p =* 0.238	0.82 (0.46, 1.45) *p =* 0.491
Model 2^b^	1.00	0.61 (0.47, 0.80) *p*<0.001	0.81 (0.62, 1.07) *p =* 0.138	0.81 (0.46, 1.45) *p =* 0.485
Model 3^c^	1.00	0.71 (0.54, 0.94) *p =* 0.015	0.79 (0.60, 1.04) *p =* 0.095	0.77 (0.43, 1.37) *p =* 0.373
Model 4^d^	1.00	0.65 (0.50, 0.85) *p =* 0.002	0.78 (0.59, 1.02) *p =* 0.071	0.72 (0.40, 1.28) *p =* 0.262
ICC for Model 1: 0.029 (0.023, 0.037)				
Vegetable consumption				
Model 1^a^	1.00	1.21 (0.92, 1.60) *p =* 0.178	0.91 (0.70, 1.20) *p =* 0.521	1.60 (0.79, 3.26) *p =* 0.191
Model 2^b^	1.00	1.12 (0.85, 1.49) *p =* 0.422	0.92 (0.70, 1.21) *p =* 0.544	1.60 (0.78, 3.27) *p =* 0.197
Model 3^c^	1.00	1.21 (0.91, 1.60) *p =* 0.196	0.90 (0.68, 1.18) *p =* 0.430	1.50 (0.74, 3.07) *p =* 0.262
Model 4^d^	1.00	1.17 (0.88, 1.56) *p =* 0.270	0.89 (0.67, 1.17) *p =* 0.391	1.45 (0.71, 2.96) *p =* 0.308
ICC for Model 1: 0.014 (0.010, 0.020)				
Fruit consumption				
Model 1^a^	1.00	0.58 (0.46, 0.73) *p*<0.001	0.73 (0.58, 0.92) *p =* 0.009	0.49 (0.29, 0.86) *p =* 0.012
Model 2^b^	1.00	0.54 (0.42, 0.68) *p*<0.001	0.73 (0.58, 0.93) *p =* 0.012	0.48 (0.28, 0.84) *p =* 0.010
Model 3^c^	1.00	0.48 (0.38, 0.61) *p*<0.001	0.74 (0.58, 0.94) *p =* 0.014	0.49 (0.28, 0.86) *p =* 0.013
Model 4^d^	1.00	0.48 (0.38, 0.61) *p*<0.001	0.74 (0.58, 0.94) *p =* 0.015	0.50 (0.29, 0.88) *p =* 0.016
ICC for Model 1: 0.007 (0.005, 0.010)				
Red meat consumption				
Model 1^a^	1.00	0.90 (0.72, 1.11) *p =* 0.312	1.04 (0.83, 1.31) *p =* 0.715	1.02 (0.62, 1.67) *p =* 0.941
Model 2^b^	1.00	0.89 (0.72, 1.11) *p =* 0.298	1.06 (0.84, 1.34) *p =* 0.605	1.02 (0.62, 1.68) *p =* 0.935
Model 3^c^	1.00	0.88 (0.71, 1.09) *p =* 0.249	1.05 (0.83, 1.32) *p =* 0.693	1.02 (0.62, 1.68) *p =* 0.940
Model 4^d^	1.00	0.90 (0.72, 1.11) *p =* 0.319	1.05 (0.84, 1.33) *p =* 0.685	1.04 (0.63, 1.71) *p =* 0.873
ICC for Model 1: 0.004 (0.002, 0.007)				
Processed meat consumption				
Model 1^a^	1.00	0.29 (0.24, 0.37) *p*<0.001	0.82 (0.63, 1.07) *p =* 0.148	0.76 (0.43, 1.32) *p =* 0.327
Model 2^b^	1.00	0.26 (0.21, 0.33) *p*<0.001	0.88 (0.67, 1.15) *p =* 0.351	0.76 (0.43, 1.34) *p =* 0.338
Model 3^c^	1.00	0.23 (0.18, 0.29) *p*<0.001	0.91 (0.70, 1.19) *p =* 0.490	0.81 (0.45, 1.44) *p =* 0.467
Model 4^d^	1.00	0.23 (0.18, 0.29) *p*<0.001	0.92 (0.70, 1.20) *p =* 0.525	0.83 (0.47, 1.48) *p =* 0.526
ICC for Model 1: 0.015 (0.011, 0.021)				
Alcohol consumption				
Model 1^a^	1.00	0.12 (0.06, 0.24) *p*<0.001	0.44 (0.29, 0.66) *p*<0.001	0.60 (0.27, 1.32) *p =* 0.204
Model 2^b^	1.00	0.10 (0.05, 0.20) *p*<0.001	0.47 (0.31, 0.71) *p*<0.001	0.56 (0.25, 1.25) *p =* 0.156
Model 3^c^	1.00	0.10 (0.05, 0.20) *p*<0.001	0.46 (0.30, 0.70) *p*<0.001	0.55 (0.24, 1.23) *p =* 0.146
Model 4^d^	1.00	0.10 (0.05, 0.20) *p*<0.001	0.46 (0.30, 0.71) *p*<0.001	0.57 (0.25, 1.28) *p =* 0.171
ICC for Model 1: 0.016 (0.011, 0.024)				
Smoking status				
Model 1^a^	1.00	3.11 (2.35, 4.12) *p*<0.001	1.18 (0.78, 1.79) *p =* 0.429	1.13 (0.45, 2.84) *p =* 0.798
Model 2^b^	1.00	2.99 (2.25, 3.98) *p*<0.001	1.06 (0.70, 1.61) *p =* 0.792	1.13 (0.44, 2.85) *p =* 0.803
Model 3^c^	1.00	2.45 (1.83, 3.26) *p*<0.001	1.06 (0.70, 1.62) *p =* 0.775	1.25 (0.49, 3.20) *p =* 0.636
Model 4^d^	1.00	2.29 (1.72, 3.06) *p*<0.001	1.08 (0.71, 1.65) *p =* 0.706	1.32 (0.52, 3.37) *p =* 0.563
ICC for Model 1: 0.037 (0.026, 0.052)				
Sleep duration				
Model 1^a^	1.00	1.93 (1.54, 2.42) *p*<0.001	1.41 (1.10, 1.82) *p =* 0.008	1.11 (0.62, 1.98) *p =* 0.728
Model 2^b^	1.00	2.00 (1.59, 2.51) *p*<0.001	1.49 (1.15, 1.93) *p =* 0.002	1.13 (0.63, 2.03) *p =* 0.677
Model 3^c^	1.00	1.70 (1.35, 2.14) *p*<0.001	1.53 (1.19, 1.98) *p =* 0.001	1.22 (0.68, 2.20) *p =* 0.502
Model 4^d^	1.00	1.60 (1.27, 2.02) *p*<0.001	1.54 (1.19, 1.99) *p =* 0.001	1.21 (0.67, 2.18) *p =* 0.522
ICC for Model 1: 0.008 (0.005, 0.013)				

***Note***. Odds ratios with 95% Confidence Intervals

^a^ Model 1: Unadjusted

^b^ Model 2: Age and gender

^c^ Model 3: Model 2 + educational qualifications, employment status, and marital status

^d^ Model 4: Model 3 + Socio-Economic Index for Areas and Accessibility/Remoteness Index of Australia

ICC: Intra-class correlation coefficient

Table 3 presents the results for the combined lifestyle index score of those of Lebanese ethnicity compared with those of Australian ethnicity. The Intra-class correlation coefficient (ICC) for Model 1 (0.008) indicates that there was no substantial variance in the lifestyle index score attributable to the Statistical Area Level 2. In Model 1, the Lebanese-born and Australian-born Lebanese ethnic groups had a lower lifestyle index score (clustering of suboptimal lifestyle behaviours) on average in comparison to those of Australian ethnicity. The Lebanese ethnic group born elsewhere had a lower lifestyle index score in comparison to the Australian ethnic group, however not statistically significant. After adjustments for socio-demographic variables were made in subsequent models, the difference in the mean lifestyle index score was further strengthened for the Lebanese-born Lebanese ethnic group and remained relatively unchanged for Australian-born Lebanese ethnic group when compared with the Australian ethnic group. The lifestyle index score for the Lebanese ethnic group born elsewhere remained relatively unchanged.

**Table 3:** Regression coefficients (95% CI) for the lifestyle index score among those of Lebanese ethnicity relative to those of Australian ethnicity.

	Australian ethnicity (ref.)	Lebanese ethnicity		
		Lebanese-born	Australian-born	Born in other countries
Model 1^a^	1.00	-0.18 (-0.33, -0.02) *p =* 0.024	-0.20 (-0.36, -0.04) *p =* 0.017	-0.04 (-0.38, 0.31) *p =* 0.840
Model 2^b^	1.00	-0.24 (-0.39, -0.09) *p =* 0.002	-0.16 (-0.32, -0.01) *p =* 0.040	-0.04 (-0.38, 0.30) *p =* 0.814
Model 3^c^	1.00	-0.32 (-0.46, -0.17) *p<*0.001	-0.17 (-0.32, -0.01) *p =* 0.034	-0.03 (-0.36, 0.31) *p =* 0.881
Model 4^d^	1.00	-0.36 (-0.51, -0.22) *p<*0.001	-0.17 (-0.32, -0.02) *p =* 0.031	-0.04 (-0.37, 0.30) *p =* 0.831
ICC for Model 1: 0.008 (0.005, 0.011)				

***Note***. Coefficients with 95% Confidence Intervals

^a^ Model 1: Unadjusted

^b^ Model 2: Age and gender

^c^ Model 3: Model 2 + educational qualifications, employment status, and marital status

^d^ Model 4: Model 3 + Socio-Economic Index for Areas and Accessibility/Remoteness Index of Australia

ICC: Intra-class correlation coefficient

## Discussion

This study aimed to examine the lifestyle behaviours of those of Lebanese ethnicity born in Lebanon, Australia, and elsewhere relative to those of Australian ethnicity. The findings of this study suggest that the lifestyle behaviours of those of Lebanese ethnicity vary by country of birth when compared to those of Australian ethnicity. While a lifestyle index score is useful for the examination of the clustering of suboptimal lifestyle behaviours among individuals, it does not provide a complete picture with respect to which key suboptimal lifestyle behaviours are most prominent [32].

The results of this study indicated that those of Lebanese ethnicity born in Lebanon had a lower lifestyle index score on average in comparison to those of Australian ethnicity. It would appear that this finding supports the ‘healthy migrant effect’, however, 36% of those of Lebanese ethnicity born in Lebanon arrived in Australia from 1975–1990 which coincided with the civil war period [23]. Thus, the mode of arrival (as potential refugees) for many Lebanese-born migrants to Australia opposes those described in the ‘healthy migrant effect’ [17]. Although there was a lower level of suboptimal lifestyle behaviours clustering among those of Lebanese ethnicity born in Lebanon, there were key differences in individual lifestyle behaviours when compared with those of Australian ethnicity. A closer examination of the individual lifestyle behaviours of those of Lebanese ethnicity born in Lebanon revealed that they were more likely to report not meeting PA guidelines, smoking, and suboptimal sleep durations when compared with those of Australian ethnicity.

The evidence to date on Australians of Lebanese ethnicity has predominantly focused on those born in Lebanon, and this evidence suggests that suboptimal lifestyle behaviours such as insufficient vegetable consumption, insufficient PA, and smoking are more prevalent among Lebanese-born adults than Australian-born adults [31, 32]. The findings from this study lend support and add strength to these earlier prevalence estimates regarding insufficient PA and smoking. Similarly, the lifestyle behaviours of smoking and insufficient PA are highly prevalent among Lebanese adults in the Lebanese context [30], which may suggest that the lifestyle behaviours of those of Lebanese ethnicity born in Lebanon are still similar to their counterparts in their origin country. Furthermore, the findings also suggest that those of Lebanese ethnicity born in Lebanon were less likely to report sitting for prolonged periods, insufficient fruit consumption, processed meat consumption, and excessive alcohol consumption, and were more likely to report suboptimal sleep durations. The lower level of alcohol consumption observed was expected and can be most likely explained by the fact that a large proportion of those of Lebanese ethnicity are of the Islamic faith [22] in which alcohol consumption is prohibited. This may be reflected by findings among adults from the Lebanese context, in which a large proportion did not report consuming alcohol within the past 12-months (59%) [30]. It is important, however, to examine differences in the health of those of Lebanese ethnicity by country of birth, as it has been shown health differs by country of birth among ethnic groups of the same origin [34, 35].

The lifestyle index score was also lower among those of Lebanese ethnicity born in Australia when compared with those of Australian ethnicity. Potential lifestyle behaviours that contributed to this lower score could be that those of Lebanese ethnicity born in Australia were less likely to report insufficient fruit and excessive alcohol consumption. Further, no differences were observed for the other lifestyle behaviours, with the exception of sleep duration. Although explicit measures of acculturation were not accounted for in this study, acculturation could potentially be a key factor in the minimal differences observed in the individual lifestyle behaviours. To elaborate, generational status is a commonly used measure of acculturation [51]. It could be argued that the lifestyle behaviours of later generation descendants of immigrants are likely to converge with those of the host population. However, the convergence of lifestyle behaviours among later generation descendants of immigrants to those of the host population do not always necessarily occur for all lifestyle behaviours [19, 20]. This was apparent in the present study for the lifestyle behaviours fruit consumption, alcohol consumption, and sleep duration among those of Lebanese ethnicity born in Australia that remained similar to those of Lebanese ethnicity born in Lebanon. These lifestyle behaviours of those of Lebanese ethnicity born in Australia, however, appear to be closer to those of Australian ethnicity, which may reflect the acculturative process.

No differences were observed in the clustering of suboptimal lifestyle behaviours among those of Lebanese ethnicity born elsewhere in comparison to those of Australian ethnicity. Those of Lebanese ethnicity born elsewhere had higher odds of reporting insufficient vegetable consumption and PA levels, and had lower odds of reporting insufficient fruit consumption. However, due to the heterogeneity on the reported countries of birth for this ‘elsewhere’ group, the findings must be interpreted with caution.

Of the Lebanese ethnic groups, those born in Lebanon were the most likely to differ in the lifestyle index and the individual lifestyle behaviours when compared with those of Australian ethnicity. A potential explanation for this could be that those of Lebanese ethnicity born in Lebanon are less acculturated, meaning that they could potentially be maintaining the lifestyle behaviours of their origin country. While it could be argued that these lifestyle behaviours are potentially maintained due to the presence of ethnic enclaves, the results of multilevel modelling suggested that no substantial variation in the lifestyle behaviours among those of Lebanese ethnicity was attributable to area of residence. Although those of Lebanese ethnicity who were born in Australia, who are later generation descendants of Lebanese immigrants, may retain some of the lifestyle behaviours of their parents, they are most likely to have lifestyle behaviours that are more closely aligned with the Australian culture. To elaborate, alcohol consumption is prohibited in Islam, a religion with a large number of Lebanese adherents [22], which could potentially explain why participants of Lebanese ethnicity born in Lebanon and Australia were less likely to report excessive alcohol consumption in comparison to those of Australian ethnicity. However, the Australian-born Lebanese ethnic group were the closer of these 2 Lebanese ethnic groups to those of Australian ethnicity in regards to excessive alcohol consumption and other lifestyle behaviours. This suggests that there could be an acculturative shift of the lifestyle behaviours of those of Lebanese ethnicity born in Australia towards those of Australian ethnicity.

### Strengths and limitations

A strength of this study is that it contributes to the limited body of evidence on the health of Australians of Lebanese ethnicity. The extensive number of variables examined in The 45 and Up Study allowed for a wide range of lifestyle behaviours and socio-demographic factors to be accounted for in this study. It is important to examine differences in health of those of Lebanese ethnicity by country of birth, as it has been shown that health differs by country of birth among ethnic groups of the same origin [34, 35]. The stratification of those of Lebanese ethnicity by country of birth, helped highlight any similarities or differences among the Lebanese ethnic groups that were potentially attributable to place of birth. Additionally, this study examined individual lifestyle behaviours in conjunction with the widely used lifestyle index [7, 8, 32, 38]. A further strength is the use of robust methods in multilevel models which allowed for the variance attributable to individual-level factors (e.g., age and gender) to be partitioned from the variance attributable to area-level factors (e.g., geographic residence) [52].

The 45 and Up Study has a focus on promoting healthy ageing by sampling those aged 45 and older, consequently the results of this study cannot be generalised to those under the age of 45. The low response rate (18%) may further limit the generalisability of the findings. The cross-sectional nature of this study only provides a snapshot of the participants’ lifestyles at one point in time and therefore changes in lifestyle behaviours overtime could not be studied. Furthermore, unmeasured factors that determine why some ethnic groups are more likely to participate in specific lifestyle behaviours than others limits causal inference. Although self-report measures are useful for collecting data on larger populations, there is potential for misreporting on various measures, including PA [53], weight, and height [54]. Moreover, as the questionnaire was only provided in English, those with limited English proficiency may not have been able to complete the survey [36]. Consequently, this could have potentially resulted in underreporting of the various lifestyle behaviours among those of Lebanese ethnicity born in Lebanon and elsewhere who may have limited English proficiency. While appropriate cut-points informed by guidelines or recommendations for distinguishing ‘optimal’ from ‘suboptimal’ lifestyle behaviours were adopted, it must be acknowledged that some of the cut-points used may not be a true reflection of what is considered ‘optimal’ and ‘suboptimal’. For example, the alcohol consumption cut-point of >14 standard drinks per week may not translate to >2 standard drinks per day and the ‘optimal’ consumption of red meat 3–4 times per week would mean that those who follow vegan or vegetarian diets would be placed in the ‘suboptimal’ category. A further limitation is the small sample sizes of the Lebanese ethnic groups, nevertheless, the results of this study provide a unique insight into the health of people of Lebanese ethnicity in Australia.

## Conclusions

This study provides a unique insight into the health of Australians of Lebanese ethnicity. While the lifestyle index suggested that there was a lower level of suboptimal lifestyle behaviour clustering among those of Lebanese ethnicity born in Lebanon and Australia in comparison to those of Australian ethnicity, the examination of the individual lifestyle behaviours showed the potential contributors to these lifestyle index scores. The findings from this study provide important evidence on the lifestyle behaviours of Australians of Lebanese ethnicity, and such evidence could potentially be utilised to develop health promotion initiatives or interventions targeting these key lifestyle behaviours. As lifestyle-related chronic diseases such as cardiovascular disease [26], T2DM [26, 28], and cancer [27] are prevalent among Lebanese immigrants, the findings of this study helps underline key lifestyle behaviours that could be targeted by health promotion service providers. Future research among those of Lebanese ethnicity could examine the impact of acculturation on the lifestyle behaviours and the contribution of such lifestyle behaviours on the risk of a range of lifestyle-related chronic diseases.

## Supporting information

S1 Fig Participant flow. (DOCX)
