# Supplementary material for: Lifestyle behaviours of Lebanese-Australians: Cross-sectional findings from The 45 and Up Study
Source: PLoS One. 2017 Jul 13;12(7):e0181217. doi: 10.1371/journal.pone.0181217 (PMC5509310; doi:10.1371/journal.pone.0181217)
Supplement: S1 Fig — (DOCX) [file pone.0181217.s001.docx]

**S1 Fig. Participant flow.**

**Final sample**

n=37,419

**Valid Statistical Area Level 2 data**

n=45,368

**Valid lifestyle index score**

n=45,368

**Participants of Australian and Lebanese ethnicity**

n=63,400

**Missing/Invalid data on at least one lifestyle behaviour**

n=18,032

**Missing/invalid socio-demographic data**

n=901

**Missing/invalid Statistical Area Level 2**

n=7,048
